# Attitudes, Practices, and Barriers of Malaysian Community Pharmacists Toward Provision of Weight Management Services

**DOI:** 10.3389/fphar.2019.00138

**Published:** 2019-02-22

**Authors:** Rohit Kumar Verma, Thomas Paraidathathu, Nur Akmar Taha, Wei Wen Chong

**Affiliations:** ^1^Department of Pharmacy Practice, School of Pharmacy, International Medical University, Kuala Lumpur, Malaysia; ^2^Faculty of Pharmacy, Universiti Kebangsaan Malaysia, Kuala Lumpur, Malaysia; ^3^Faculty of Health and Medical Sciences, Taylor’s University, Subang Jaya, Malaysia; ^4^Faculty of Pharmacy, Cyberjaya University College of Medical Sciences, Cyberjaya, Malaysia

**Keywords:** attitude, practices, barriers, pharmacists, obesity, Malaysia

## Abstract

In Malaysia, sharp increment in the prevalence of obesity over the last four decades has been documented. Community pharmacists (CPs) are strategically placed to tackle obesity by providing weight managements services (WMS) to general public. This study assessed the attitudes, practices and perceived barriers of Malaysian CPs to the provision of WMS. A cross-sectional, descriptive survey was conducted, and responses related to attitudes, practices and perceived barriers of CPs were collected using five-point Likert scale. A total of 550 pharmacists who worked across six states of Malaysia (Selangor, Federal Territory of Kuala Lumpur, Pulau Pinang, Johor, Sabah, and Melaka) participated in this study. Most of the CPs strongly agreed that over eating (*n* = 312, 56.7%) and sedentary lifestyles (*n* = 297, 54.0%) contribute to obesity and overweight. Most of them also strongly agreed that exercise training is an effective weight reduction strategy (*n* = 285, 51.9%), but they were generally not in favor of surgery (*n* = 231, 42% disagreed/strongly disagreed). CPs generally perceived barriers related to a lack of staff to provide WMS (*n* = 308, 56.0% agreed/strongly agreed) and ethical and legal issues associated with sales of products/drugs for obesity management (*n* = 285, 51.9% agreed/strongly agreed). Sociodemographic and practice characteristics such as age group, type of pharmacy, highest education qualification, and employment status of CPs influenced the attitudes, practices and perceived barriers associated with WMS. In terms of age, CPs who were aged less then 30 years expressed significantly stronger agreement that medication adherence is beneficial for weight loss compared to those CPs between 41–50 years. Additionally, CPs who were pharmacy owners provided significantly more frequent BMI measurement and patient information materials as part of their weight management practices compared to CPs who worked as a part timer/locum. This study could be taken as a baseline study on Malaysian CPs’ perceptions on WMS.

## Introduction

Obesity is a global concern whereby the global population is not merely expanding in numbers, but also in weight. According to the definition of WHO, for adults aged 18 years old and over, overweight is defined as a BMI of 25 kg/m^2^ and above, while obesity is defined as a BMI of 30 kg/m^2^ and above (GHO, 2017). As per WHO, the prevalence of obesity worldwide has nearly tripled between the years 1975 and 2016. In 2016, more than 1.9 billion adults were overweight. Among them, more than 650 million were identified as being obese, corresponding to 13% of the world’s adult populations (11% of men and 15% of women). The obesity outbreak has also hit growing economies such as Malaysia. In Malaysia, sharp increment in the prevalence of obesity over the last four decades has been clearly documented. In 1975, about 1.4% of Malaysian adult population was obese, but the figure has since raised to 15.6% in 2016, with more than 10 times increase in the prevalence of obesity (GHO, 2017). Studies in Malaysia have also shown that this increase in prevalence of obesity also affects children and adolescents (Mohammed Nawi and Che Jamaludin, 2015; Sabramani et al., 2015;Se Xian et al., 2016).

Pharmacists being important members of the healthcare team, have an important role in caring for overweight and obese patients. The potential role of pharmacists in weight management, especially those working in the community settings, is great. CPs are approached by patients from all walks of life in the community, which therefore presents ample opportunities for them to involve in weight management. They can assist patients with decision-making regarding pharmacological weight loss approaches, increase compliance and support for changes in lifestyle, as well as caution patients on the dangers of improper use of the growing numbers of over-the-counter weight loss products promising quick benefits (Krska et al., 2010; Kerrigan et al., 2011; Verma et al., 2013; Kumar Verma and Paraidathathu, 2014).

Although a review of studies in which CPs provided weight management alone or in a team showed that studies in this area were limited (*n* = 6), there were, however, some successful outcomes reported (O’Neal and Crosby, 2014). In an Australian study, the authors investigated the perception of CPs, pharmacy assistants and women on CPs’ involvement in weight management. It was found that CPs, pharmacy assistants and women unanimously perceived CPs to assume an important role in weight management. They recommended the use of pharmacy-specific weight management educational resources that were readily available to pharmacy staff and consumers (Fakih et al., 2016). Another group of researchers evaluated the outcomes of a community pharmacy based weight management program in Australia, ‘AHLP,’ which focused on three areas: diet, physical activity and behavioral change. The AHLP was well received by the participants, with 32% of them achieving ≥5% weight loss from baseline, suggesting the potential of such evidence-based patient-centered community pharmacy based weight management program (Um et al., 2015). Similar findings were reported in a study from England which showed significant reductions in weight and waist circumference among patients who participated in a community pharmacy weight management program (Boardman and Avery, 2014). A study in Thailand also reported that CPs were able to improve both eating behavior and knowledge about weight management among obese patients (Phimarn et al., 2013). Nevertheless, despite its positive outcomes, CPs have also identified several barriers in providing weight management services, including lack of time, lack of patient demand/expectations and lack of reimbursement (O’Donnell et al., 2003).

In view of successful outcomes reported worldwide, one of the approaches that could be taken to tackle obesity in Malaysia would be the involvement of CPs in providing WMS. According to previous surveys conducted, about 75–90% of the CPs in Malaysia participated in the delivery of extended pharmacy services, including WMS (Hassali et al., 2009; Ooi et al., 2016). Furthermore, in our previous study, we have found that the general public in Malaysia supported the weight management services provided by CPs, however, only few respondents agreed that they are utilizing these services (Verma et al., 2018). Thus, there is still immense potential to expand this specialized pharmacy service. Prior to involving CPs in a larger scale, it is essential to understand their attitudes, practices and perceived barriers in providing WMS. Hence this study was undertaken to assess the attitudes, practices and perceived barriers of Malaysian CPs in the delivery of WMS.

## Materials and Methods

### Study Design

A descriptive, cross sectional survey was conducted to explore Malaysian CPs’ attitudes, practices, and barriers with their current provision of WMS in community pharmacy.

### Study Participants and Sampling

This survey was conducted on Malaysian registered CPs from six different states of Malaysia (Selangor, Federal Territory of Kuala Lumpur, Pulau Pinang, Johor, Sabah, and Melaka), whereby CPs with minimum 2 years of community pharmacy practice experience and who were willing to participate were enrolled in the study. Non-registered pharmacists and pharmacy assistants were excluded. Convenience sampling method was adopted in order to obtain a satisfactory response.

The sample size calculated using Krejcie and Morgan formula (Krejcie and Morgan, 1970) was 357 as given below. In order to achieve a useable sample size of 357, 714 CPs were targeted with an assumed response rate of 50%.

$$s\;=\;X^{2}\;NP\;(1\;-\;P)\;÷\;d^{2}\;(N\;-\;1)\;+\;X^{2}\;P\;(1\;-\;P)$$

*s* = required sample size.
*X*^2^ = the table value of chi-square for 1 degree of freedom at the desired confidence level (3.841).
*N* = the population size (5000).
*P* = the population proportion (assumed to be 0.50 since this would provide the maximum sample size).
*d* = the degree of accuracy expressed as a proportion (0.05).

### Study Instrument/Questionnaire

A self-administered questionnaire was developed after a thorough literature review on the relevant published studies (Dastani et al., 2004; Hassali et al., 2009; Newlands et al., 2011; Fakih et al., 2015; Ooi et al., 2016). An initial version of the questionnaire was subjected to content and face validity by experts (five CPs and seven academicians) who had 10 or more years of experience in the field of community pharmacy practice/community pharmacy research in Malaysia. The experts verified the appropriateness of questions and their readability. They also reviewed if each question in the questionnaire had a logical link with the objectives of the study. Pre-testing on the survey instrument was conducted among 49 CPs. Cronbach’s alpha of 0.714 was obtained for attitude and practice components of the survey instrument, while Cronbach’s alpha of 0.732 was obtained for the barriers component of the survey instrument, indicating good reliability of the questionnaire.

The final version of the questionnaire consisted of 49 statements/questions and responses were given through a five-point Likert scale. It comprised of four sections:

(i) The characteristics and sociodemographic data of CPs, including their gender, age, ethnicity, highest qualification, employment status, type and location of pharmacy where they were working;
(ii) CPs’ attitudes regarding obesity and overweight, including on causes of overweight and obesity, effectiveness of overweight and obesity management and their confidence toward overweight and obesity management (scored using a five-point Likert scale with score of 1 for strongly disagree, 2 for disagree, 3 for neutral, 4 for agree, and 5 for strongly agree);
(iii) The weight management practices of CPs, including anthropometric/physiological services, advises and sale of products. The level of frequency of involvement in different weight management practices were scored using a five-point Likert scale (with score of 1 for never, 2 for rarely, 3 for occasionally, 4 for often, and 5 for always);
(iv) The barriers CPs perceived in providing WMS in community pharmacies (scored using a five-point Likert scale with score of 1 for strongly agree, 2 for agree, 3 for neutral, 4 for disagree, and 5 for strongly disagree).

### Data Collection

Data was collected by face to face distribution of the questionnaires to CPs during events/training sessions organized by MPS in different states of Malaysia. In states where there was no such event, the questionnaires were distributed by face to face in community pharmacies.

### Ethics Statement

Ethical approval (NF-016-14) for this study was obtained from the Ethics Committee of Universiti Kebangsaan Malaysia, Kuala Lumpur, Malaysia. Participation in the study was voluntary and written consent was obtained from the respondents prior to their participation.

### Statistical Analysis

The data were analyzed using SPSS^®^ program, version 23.0 for Windows^®^ (IBM, Armonk, New York, NY, United States). Descriptive statistics of frequency and percentage and median values with IQR were applied for characteristics of the respondents and the response for each statement in the study instrument. Reverse coding was done for negatively worded statements. As it was observed that the data were not normally distributed, associations between respondents’ characteristics and the mean ranks of their attitudes, practices, and barriers toward WMS were evaluated using Mann–Whitney *U* and Kruskal–Wallis tests of association. Dunn’s *post hoc* multiple comparison tests with Bonferroni correction were performed following a statistically significant Kruskal–Wallis test. All statistical tests were performed at an *a priori* significance level of *p* = 0.050.

## Results

### Demographic Characteristics

Out of 714 CPs to whom the questionnaires were distributed, 563 completed questionnaires were received (78.9% response rate). Of these, 13 questionnaires were excluded due to inappropriate filling. Final results were based on the analysis on 550 respondents. The characteristics of the respondents are shown in Table 1.

**Table 1:** Sociodemographic and practice characteristics of the respondents.

Characteristics	*N* (%)
**Gender**	
Female	361 (65.6)
Male	189 (34.4)
**Age range (years)**	
Under 30	173 (31.5)
30–40	245 (44.5)
41–50	83 (15.1)
51–60	38 (6.9)
61–70	10 (1.8)
More than 70	1 (0.2)
**Ethnicity**	
Malay	89 (16.2)
Chinese	421 (76.5)
Indian	37 (6.7)
Others	3 (0.5)
**Highest educational qualification**	
Bachelor’s degree	473 (86.0)
Master’s degree	76 (13.8)
Doctoral degree	1 (0.2)
**Type of pharmacy**	
Independent pharmacy	342 (62.2)
Chain pharmacy	208 (37.8)
**Employment status of pharmacist**	
Part time employee/locum pharmacist	94 (17.1)
Permanent employee	286 (52)
Pharmacy owner	159 (28.9)
Details not available	11 (2)
**Location of community pharmacy (states)**	
Penang	50 (9.1)
Johor	21 (3.8)
Melaka	21 (3.8)
Sabah	78 (14.2)
Federal Territory Kuala Lumpur	219 (39.8)
Selangor	161 (29.3)

### Attitudes Regarding Obesity and Weight Management

CPs’ attitudes regarding obesity and overweight are shown in Table 2. CPs generally agreed/strongly agreed with every cause of overweight and obesity listed (ranged from 55.1 to 96.3%), and in particular strongly agreed that over eating (*n* = 312, 56.7%) and sedentary lifestyle (*n* = 297, 54.0%) contribute to obesity and overweight. Most of the CPs also strongly agreed that exercise training is an effective weight reduction strategy (*n* = 285, 51.8%), but they were generally not in favor of surgery (*n* = 231, 42% disagreed/strongly disagreed) as an effective intervention for weight management. Overall, the respondents regarded themselves to be well-prepared to manage overweight and obese patients (*n* = 364, 66.2% agreed/strongly agreed). Nevertheless, they generally agreed that CPs alone cannot manage overweight and obesity effectively (*n* = 395, 71.8% agreed/strongly agreed).

**Table 2:** Community pharmacist’s attitude regarding obesity and overweight.

In your opinion what are the causes of	SD	D	N	A	SA	Median
obesity and overweight?	*n* (%)	*n* (%)	*n* (%)	*n* (%)	*n* (%)	(IQR)
**A. Attitude regarding causes of obesity and overweight:**						
Over eating	8 (1.5)	6 (1.1)	6 (1.1)	218 (39.6)	312 (56.7)	5 (4-5)
Sedentary life style	9 (1.6)	2 (0.4)	10 (1.8)	232 (42.2)	297 (54.0)	5 (4-5)
Eating at restaurants	15 (2.7)	121 (22.0)	111 (20.2)	231 (42.0)	72 (13.1)	4 (3-4)
High fat diet and repeat diet	8 (1.5)	18 (3.3)	36 (6.5)	268 (48.7)	220 (40.0)	4 (4-5)
Genetic factors	6 (1.1)	36 (6.5)	73 (13.3)	350 (63.6)	85 (15.5)	4 (4-4)
Hormonal problems	5 (0.9)	16 (2.9)	69 (12.5)	378 (68.7)	82 (14.9)	4 (4-4)
Consumption of alcohol	5 (0.9)	34 (6.2)	91 (16.5)	323 (58.7)	97 (17.6)	4 (4-4)
Stress, anxiety, depression	3 (0.5)	41 (7.5)	94 (17.1)	326 (59.3)	86 (15.6)	3 (4-4)
Psychological problems	4 (0.7)	45 (8.2)	118 (21.5)	306 (55.6)	77 (14.0)	4 (3-4)
Family culture/environment	4 (0.7)	27 (4.9)	60 (10.9)	355 (64.5)	104 (18.9)	4 (4-4)

**B. Attitude regarding effectiveness of obesity and overweight management:**				

I think weight loss surgery is effective.	55 (10.0)	176 (32.0)	133 (24.2)	162 (29.5)	24 (4.4)	3 (2-4)
I think dietary supplements are effective.	8 (1.5)	66 (12.0)	107 (19.5)	329 (59.8)	40 (7.3)	4 (3-4)
I feel exercise training is effective.	0 (0.0)	3 (0.5)	11 (2.0)	251 (45.6)	285 (51.8)	5 (4-5)
In my opinion medications are effective.	14 (2.5)	53 (9.6)	80 (14.5)	358 (65.1)	45 (8.2)	4 (3-4)
Counseling by dieticians is effective.	0 (0.0)	6 (1.1)	70 (12.7)	363 (66.0)	111 (20.2)	4 (4-4)
Patient medication adherence is beneficial	1 (0.2)	17 (3.1)	65 (11.8)	346 (62.9)	121 (22.0)	4 (4-4)
Effective treatment of obesity requires teamwork amongst health professionals.	0 (0.0)	12 (2.2)	21 (3.8)	288 (52.4)	229 (41.6)	4 (4-5)

**C. Attitude toward confidence in obesity and overweight management:**				

Community pharmacist should be role models to manage overweight and obesity.	1 (0.2)	11 (2.0)	34 (6.2)	354 (64.4)	150 (27.3)	4 (4-5)
I feel I am well prepared to manage overweight and obese patients	3 (0.5)	37 (6.7)	146 (26.5)	298 (54.2)	66 (12.0)	4 (3-4)
Community pharmacists alone cannot manage obesity and overweight effectively	17 (3.1)	78 (14.2)	60 (10.9)	318 (57.8)	77 (14.0)	4 (3-4)
Training programs like “My Weight My Health” (MWMH) will help to improve my confidence.	4 (0.7)	11 (2.0)	79 (14.4)	330 (60.0)	126 (22.9)	4 (4-4)

*SD, strongly disagree; D, disagree; N, neutral; A, agree; SA, strongly agree.*

Employment status of CPs was noted to be significantly associated with the views that dietary supplements and medications are effective for weight loss management [H(3) = 13.748, *p* = 0.003 and H(3) = 13.703, *p* = 0.003, respectively]. A Bonferroni correction at a 0.05 level of significance reported that CPs who owned their pharmacies expressed significantly stronger agreement to the view that dietary supplements and medications (mean rank = 299.13 and mean rank = 281.32, respectively) are effective compared to those who worked as a part timer/locum (mean rank = 234.37 and mean rank = 229.68, respectively) (*Z* = 3.554, *p* = 0.002 and *Z* = 2.944, *p* = 0.019, respectively). A Bonferroni correction at a 0.05 level of significance also reported that CPs who worked as permanent staff (mean rank = 287.89) expressed significantly stronger agreement to the view that medications are effective compared to those who worked as a part timer/locum (mean rank = 229.68) (*Z* = 3.632, *p* = 0.002).

A significant association was also found between age groups and CPs’ perception that patient’s medication adherence is beneficial [H(5) = 14.854, *p* = 0.011]. A Bonferroni correction at a 0.05 level of significance revealed that CPs who were aged less than 30 years (mean rank = 299.72) expressed significantly stronger agreement toward this perception compared to those who were aged between 41 and 50 years (mean rank = 261.92) (*Z* = 3.503, *p* = 0.007). In addition, it was observed that CPs who were working in a chain pharmacy (mean rank = 293.42) expressed significantly stronger agreement toward this perception compared to those who were working in an independent pharmacy (mean rank = 264.60) (*U* = 31840.50, *p* = 0.016).

In terms of confidence toward the management of obesity and overweight, CPs who were working in an independent pharmacy (mean rank = 288.06) expressed significantly stronger agreement that they felt themselves to be well-prepared to manage overweight and obese patients compared to those who were working in a chain pharmacy (mean rank = 254.86) (*U* = 33655.00, *p* = 0.009).

### Weight Management Practices

Current weight management practices of CPs are shown in Table 3. The CPs reported that they generally often/always provided various services related to anthropometrical and physiological measurements (ranged from 52.4 to 90.8%). They also reported that they often/always provided patients with advice on physical activity (*n* = 510, 92.8%) and healthy eating (*n* = 515, 93.7%), as well as selling weight loss products (*n* = 481, 87.5%) and weight loss drugs (*n* = 379, 69%) as part of their weight management practices.

**Table 3:** Current weight management practices.

I provide the following WM services in the pharmacy						
A. Anthropometric/physiological	Never	Rarely	Occasionally	Often	Always	Median
	*n* (%)	*n* (%)	*n* (%)	*n* (%)	*n* (%)	(IQR)
Measurement of weight	27 (4.9)	56 (10.2)	20 (3.6)	309 (56.2)	138 (25.1)	4 (4-5)
Measurement of height	58 (10.5)	100 (18.2)	35 (6.4)	268 (48.7)	89 (16.2)	4 (2-4)
Calculation of body mass index (BMI)	37 (6.7)	47 (8.5)	19 (3.5)	325 (59.1)	122 (22.2)	4 (4-4)
Measurement of waist/hip circumference	59 (10.7)	127 (23.1)	58 (10.5)	225 (40.9)	81 (14.7)	4 (2-4)
Measurement of body fat percentage	63 (11.5)	132 (24.0)	67 (12.2)	210 (38.2)	78 (14.2)	4 (2-4)
Measurement of blood pressure	23 (4.2)	12 (2.2)	16 (2.9)	337 (61.3)	162 (29.5)	4 (4-5)
Measurement of blood glucose	27 (4.9)	11 (2.0)	18 (3.3)	330 (60.0)	164 (29.8)	4 (4-5)
Measurement of blood cholesterol	42 (7.6)	43 (7.8)	30 (5.5)	312 (56.7)	123 (22.4)	4 (4-4)
**B. Advise and sale of the weight management products and services**						
Advice on physical activity to achieve weight loss.	6 (1.1)	17 (3.1)	17 (3.1)	321 (58.4)	189 (34.4)	4 (4-5)
Advices on healthy eating to achieve weight loss.	5 (0.9)	17 (3.1)	13 (2.4)	310 (56.4)	205 (37.3)	4 (4-5)
Sale of weight loss products other than drugs (e.g., dietary aids, supplements).	7 (1.3)	22 (4.0)	40 (7.3)	363 (66.0)	118 (21.5)	4 (4-4)
Sale of weight loss drugs.	29 (5.3)	77 (14.0)	65 (11.8)	305 (55.5)	74 (13.5)	4 (3-4)
Distribution of patient information materials (e.g., leaflets, popular diet books and video CD’s).	40 (7.3)	96 (17.5)	77 (14.0)	263 (47.8)	74 (13.5)	4 (3-4)
Referral to doctors and/or dieticians.	35 (6.4)	85 (15.5)	76 (13.8)	284 (51.6)	70 (12.7)	4 (3-4)

A significant association was reported between the CP’s highest educational qualification and the frequency of weight measurement [H(2) = 8.181, *p* = 0.017]. A Bonferroni correction at a 0.05 level of significance noted that the CPs who were holding a bachelor’s degree (mean rank = 282.51) provided weight measurement significantly more frequent compared to those who were holding a master’s degree (mean rank = 232.13) (*Z* = 2.858, *p* = 0.013).

A significant association was observed between the age group and the employment status of the CP with the frequency of height measurement [H(5) = 22.574, *p* = 0.001 and H(3) = 16.102, *p* = 0.001, respectively]. A Bonferroni correction at a 0.05 level of significance revealed that the CPs who were aged 41–50 years (mean rank = 322.34) and 51–60 years (mean rank = 324.12), provided height measurement significantly more frequent compared to those who were aged less than 30 years (mean rank = 243.50) (*Z* = 3.969, *p* = 0.001 and *Z* = 3.039, *p* = 0.036, respectively). Moreover, a Bonferroni correction at a 0.05 level of significance noted that the CPs who owned their pharmacies (mean rank = 309.69) provided height measurement significantly more frequent compared to those who worked as a part timer/locum (mean rank = 242.88) and those who worked as permanent staff (mean rank = 263.46) (*Z* = 3.465, *p* = 0.003 and *Z* = 3.149, *p* = 0.010, respectively). In addition, CPs who were working in an independent pharmacy (mean rank = 297.44) provided height measurement significantly more frequent than those who were working in a chain pharmacy (mean rank = 236.72) (*U* = 27472.50, *p* = 0.001).

The highest educational qualification and employment status of CPs were significantly associated with the frequency of calculation of BMI [H(2) = 8.348, *p* = 0.015 and H(2) = 8.546, *p* = 0.036, respectively]. CPs who were holding a Bachelor’s degree (mean rank = 281.97) and those who owned their pharmacies (mean rank = 291.13), calculated BMI significantly more frequent than those who were holding a master’s degree (mean rank = 231.87) and those who worked as a part timer/locum (mean rank = 242.69) (*Z* = 2.889, *p* = 0.012 and *Z* = 2.651, *p* = 0.048, respectively). Furthermore, CPs who were working in an independent pharmacy (mean rank = 289.97) calculated BMI significantly more frequent than those who were working in a chain pharmacy (mean rank = 250.46) (*U* = 34052.00, *p* = 0.001).

A significant association was found between age group and employment status of CPs with the frequency of body fat percentage measurement [H(5) = 23.266, *p* = 0.001 and H(2) = 19.189, *p* = 0.001, respectively]. CPs whose age was between 41 and 50 years (mean rank = 309.86) and whose age was between 51 and 60 years (mean rank = 350.17), provided significantly more frequent body fat percentage measurement than those whose age was less than 30 years (mean rank = 243.50) (*Z* = 3.335, *p* = 0.013 and *Z* = 3.971, *p* = 0.001, respectively). CPs who owned their pharmacies (mean rank = 313.71) also provided significantly more frequent body fat percentage measurement than those who worked as a part timer/locum (mean rank = 252.66) and those who worked as permanent staff (mean rank = 257.73), after a Bonferroni correction at a 0.05 level of significance (*Z* = 3.072, *p* = 0.013 and *Z* = 3.702,*p* = 0.001, respectively).

A significant association was found between employment status of CPs and the frequency of distribution of patient information materials [H(2) = 8.320, *p* = 0.040]. After a Bonferroni correction at a 0.05 level of significance, it was observed that CPs who owned their pharmacies (mean rank = 301.05) distributed patient information materials significantly more frequent than those who worked as a part timer/locum (mean rank = 249.40) (*Z* = 2.664, *p* = 0.046). In addition, CPs who were working in an independent pharmacy (mean rank = 287.44) distributed patient information materials significantly more frequent than those who were working in a chain pharmacy (mean rank = 254.61) (*U* = 31223.50, *p* = 0.012).

### Barriers to Provision of WMS

Barriers to the provision of WMS in community pharmacies are shown in Table 4. CPs generally perceived barriers related to a lack of staff to provide WMS (*n* = 308, 56.0% agreed/strongly agreed), ethical and legal issues associated with sales of products/drugs for obesity management (*n* = 285, 51.9% agreed/strongly agreed) and lack of patient willingness to utilize WMS in pharmacy (*n* = 266, 48.3% agreed/strongly agreed).

**Table 4:** Barriers to the provision of WMS in community pharmacies.

Perceived barriers:	SD	D	N	A	SA	Median
	*n* (%)	*n* (%)	*n* (%)	*n* (%)	*n* (%)	(IQR)
Patients are not willing to avail the weight management services in pharmacy.	13 (2.4)	144 (26.2)	127 (23.1)	230 (41.8)	36 (6.5)	3 (2-4)
I am very busy to provide WM services in my pharmacy.	37 (6.7)	275 (50.0)	82 (14.9)	142 (25.8)	14 (2.5)	4 (4-4)
I would require extra payment to provide weight management services.	35 (6.4)	190 (34.5)	148 (26.9)	149 (27.1)	28 (5.1)	3 (3-4)
I am apprehensive regarding ethical and legal issues related to products/drugs used in obesity management.	13 (2.4)	106 (19.3)	146 (26.5)	250 (45.5)	35 (6.4)	2 (2-3)
I do not wish to provide weight management services.	88 (16.0)	343 (62.4)	88 (16.0)	23 (4.2)	8 (1.5)	4 (4-4)
More staff would be needed to provide weight management services.	13 (2.4)	124 (22.5)	105 (19.1)	280 (50.9)	28 (5.1)	2 (2-3)
Providing weight management services in my pharmacy will increase my workload.	23 (4.2)	162 (29.5)	114 (20.7)	227 (41.3)	24 (4.4)	3 (2-4)
My pharmacy does not have a private consultation room.	65 (11.8)	242 (44.0)	43 (7.8)	177 (32.2)	23 (4.2)	4 (4-4)
My pharmacy doesn’t have relevant machines/equipment to provide weight management services.	66 (12.0)	281 (51.1)	46 (8.4)	139 (25.3)	18 (3.3)	4 (4-4)
I believe that providing weight management services is not a part of my responsibilities/duties.	123 (22.4)	335 (60.9)	50 (9.1)	33 (6.0)	9 (1.6)	4 (4-4)

*SD, strongly disagree; D, disagree; N, neutral; A, agree; SA, strongly agree.*

A significant association was reported between employment status of CP and the perceived barrier of requiring extra payment to provide WMS [H(2) = 13.709, *p* = 0.003]. A Bonferroni correction at a 0.05 level of significance noted that CPs who were permanent staff (mean rank = 295.46) expressed significantly stronger agreement toward this barrier compared to those who owned their pharmacies (mean rank = 248.50) (*Z* = 3.116, *p* = 0.011).

On the other hand, CPs who were working in a chain pharmacy (mean rank = 291.27) expressed significantly stronger agreement toward the perceived barrier of a lack of private consultation room than those who were working in an independent pharmacy (mean rank = 265.07) (*U* = 32079.00, *p* = 0.045).

## Discussion

To the best of the authors’ knowledge, this was the first study which aimed to explore the attitudes, practices and perceived barriers of Malaysian CPs in the delivery of WMS. CPs agreed with commonly known contributory factors to obesity such as over eating and sedentary lifestyle, and that lifestyle approaches such as exercise are an effective way of weight reduction. Our findings indicated that many of the CPs were currently providing WMS, although they also reported the presence of multiple barriers such as lack of staff and ethical issues in the provision of these services. In addition, several demographic characteristics were associated with CP’s attitudes, practices and perceived barriers related to WMS.

### Attitudes Regarding Obesity and Weight Management

The study findings revealed that majority of CPs regarded over eating and sedentary lifestyles to be the main contributory factors to overweight and obesity, and that exercise training to be the most effective intervention. Through increasing energy expenditure, regular exercise has been shown to be one of the best predictors of successful weight maintenance (French et al., 2001; Shaw et al., 2006; Stice et al., 2008; Thorogood et al., 2011). There is also abundant evidence that improved fitness through regular physical activity reduces cardiovascular morbidity and mortality in overweight individuals (Bouchard et al., 1993; McInnis, 2000; Shaw et al., 2006). However, there was a significant proportion of CPs who believed that weight loss surgery is not effective. The emphasis on exercise and weight loss medicines may indicate that CPs see a role for them to be involved in these approaches to weight management, however, they need to be aware that bariatric surgery may be an effective option for severely obese patients so that they would be able to refer suitable patients to physicians.

The CPs in this study generally regarded themselves to be well equipped for the delivery of WMS. This was in line with previous international findings where CPs generally expressed positive views on their role in weight management, and that they held a unique position compared to other community-based healthcare providers, by virtue of their ability to have regular contact with patients due to prescription dispensing, and patients are more comfortable talking to CPs than to general practitioners (Um et al., 2014; Fakih et al., 2016; Gray et al., 2016). Majority of CPs in our study also perceived that training programs would help in building confidence toward the provision of WMS. Within published literature, studies that reported perceived lack of knowledge among CPs as a barrier to the provision of WMS also expected an improvement in this perception as well as self-confidence with proper training (O’Donnell et al., 2003; Dastani et al., 2004; Newlands et al., 2011; Sarayani et al., 2012).

In our study, most of the CPs felt that the problem of overweight and obesity could not be managed by CPs alone and requires teamwork amongst health professionals. In fact, among the Australian consumers, the ideal community pharmacy based WMS would be predominantly a collaborative approach where all relevant health care professionals contributed in their own field of expertise (Um et al., 2014). Therefore, an integrated healthcare approach to weight management in community pharmacy is desirable where the CP is expected to be the central professional who coordinates with other healthcare professionals, including nutritionists, dieticians and exercise physiologists. Currently, some community pharmacies in Malaysia hire dietitians and nutritionists as part of their staff, and they could prove valuable in the expansion of WMS to incorporate a multidisciplinary approach in the future.

### Weight Management Practices

It was encouraging to find that majority of the CPs in our study reported providing extended services that help in weight management, including anthropometric and physiological measurements. Anthropometric measurements such as body weight, height, BMI, waist/hip circumference, and body fat percentage would help in proper classification and monitoring of patient’s weight status at baseline and during follow-ups. Since obesity is a major cardiovascular risk factor, screening for cardiovascular risk with physiological measurements of blood pressure, blood glucose, and blood cholesterol would help in the detection of any of these abnormalities at baseline and during follow-ups in order to allow proper referral to the physicians for treatment of these comorbidities or to allow escalation of weight loss therapy with pharmacological approach.

It was also encouraging that higher proportion of CPs reported that they often/always provided advices on physical activity and healthy eating. Comprehensive lifestyle intervention comprising of diet, exercise, and behavioral modification is important in the management of overweight and obesity. With plethora of weight loss products marketed, CPs would also find themselves on the receiving end of questions from patients wishing to purchase these products. CPs should highlight the lack of efficacy data of some of the weight loss products to the patients at the point of purchase. Patients who insist on taking weight loss products, including traditional and herbal medicines, should be encouraged to purchase products that have undergone content verification (Verma et al., 2018).

The majority of CPs in our study also often/always distributed patient information materials as part of their weight management practices. These materials are beneficial in engaging and motivating the patient to promote better understanding and positive behavior change. Indeed, in an Australian study, CPs, pharmacy assistants and women perceived that educational resources were needed to assist them in weight management, especially those developed from trustworthy sources without conflict of interest. Patient information materials such as brochures, pamphlets, and self-care fact cards distributed in community pharmacies were desirable (Fakih et al., 2016).

### Barriers to Provision of WMS

The most common barrier encountered by CPs in our study was that more staff would be required to provide WMS. This barrier was also encountered by Scottish CPs, with similar proportion of Scottish CPs who agreed/strongly agreed with this barrier as Malaysian CPs (59.7% versus 56.0%) (Newlands et al., 2011). In Malaysia, community pharmacies often operate with a single CP. The situation is complicated by the fact that most community pharmacies in Malaysia have a high staff turnover rate, where CPs face difficulties in recruiting and retaining auxiliary staff (Kho et al., 2017). In addition, proper training of pharmacy staff is required before they are competent to provide evidence-based advice to pharmacy consumers regarding over-the-counter weight loss products available in pharmacy.

In addition, more than half of the CPs reported being apprehensive about ethical and legal issues associated with products/drugs used in obesity management. The ethical issues surrounding the sales of weight loss products have been documented from the consumers’ side within the published literature. There were consumers who voiced out concerns that CPs’ advices on weight management may be biased in order to profit from selling a weight loss product (Weidmann et al., 2012; Um et al., 2014). For instance, Australian community pharmacists were subjected to close scrutiny, where criticism from consumers emerged in public social media with regards to the perceived conflicts of interest of CPs selling weight loss products to increase their net revenue (Um et al., 2014). For legal issues, the absence of dispensing separation may have limited the opportunity of CPs to dispense and counsel patients who are prescribed with weight loss drugs, and may have diverted a large part of their responsibilities in WMS to the supply of over-the-counter weight loss products.

Many CPs also felt that patients were reluctant to avail WMS at the community pharmacy. A similar barrier was cited by Scottish CPs who felt that local community did not request for WMS in the community pharmacy and did not recognize that community pharmacies could provide WMS to achieve weight loss (Newlands et al., 2011). This situation could be due to the fact that many community pharmacies did not advertise WMS publicly unlike commercial slimming centers. Indeed, other studies have indicated that community pharmacies and CPs were the least cited location and health care provider, respectively, as first point of contact for advice on weight management (Um et al., 2014). In our previous study involving the general public, only 26.6% of respondents ranked pharmacists as their preferred first or second line of consultation for weight reduction (Verma et al., 2018). In addition, although the majority of respondents supported the role of CPs in providing WMS, only a small percentage reported utilizing these services (Verma et al., 2018). A lack of patient demand was also perceived to be a barrier for other pharmacy-related services in Malaysia, such as tobacco cessation service (Taha and Guat Tee, 2015).

In our study, close to one-third of the CPs surveyed agreed/strongly agreed that they wanted additional payment to provide WMS. The issue of payment has also been noted as a barrier among the general public in our previous study; with majority of the respondents preferring WMS to be provided without any additional cost (Verma et al., 2018). Quantitative studies in United States, United Kingdom, and Kuwait have also revealed this concerning issue where a half to two-thirds of CPs perceived the lack of financial reimbursement in constituting a barrier to provide WMS (O’Donnell et al., 2003; Hassali et al., 2009; Newlands et al., 2011). Profit for WMS in community pharmacy in most cases remains predominantly linked to weight loss products, at least in Malaysia currently, and therefore provides little incentive for the philosophical shift toward a more service-focused model of care. A study in Australia, found that if the WMS provided is properly remunerated, it would facilitate the provision of WMS such that CPs would be more proactive (Berbatis et al., 2003). Therefore, appropriate remuneration, for example, from the government, insurance or private payment, to the delivery of WMS is considered vital to cover the extra workload involved in administering the service, particularly the time of CP, which could potentially become a key facilitator for this service.

### Impact of Sociodemographic and Practice Characteristics

This study found that CPs who were pharmacy owners provided significantly more frequent BMI measurement and patient information materials as part of their weight management practices compared to CPs who worked as a part timer/locum, and they provided significantly more frequent height measurement and body fat percentage measurement than both the CPs who worked as permanent staff and those who worked as a part timer/locum. Our observation could be justified by the fact that pharmacy owners may have more authority over their practice environment to implement and provide WMS as desired. Therefore, they may be able to provide more individualized services to their customers.

In our study, CPs who were the pharmacy owner expressed significantly higher level of agreement toward the effectiveness of dietary supplements and medicines than CPs who worked as a part timer/locum. This may be related to the absence of remuneration for providing WMS in community pharmacy, thus profit for WMS in most cases remains predominantly linked to weight loss products. In fact, CPs working as permanent staff who may be financially incentivized based on sales target also demonstrated significantly higher level of agreement toward the effectiveness of weight loss medicines compared to CPs who worked as a part timer/locum. Hence, it was also not surprising to observe that CPs who were working as permanent staff perceived more strongly the barrier related to a lack of remuneration for WMS than pharmacy owners, since they may not be financially incentivized if the WMS provided does not associate with the sales of weight loss products.

It was also observed in our study that CPs working in an independent pharmacy demonstrated significantly stronger agreement that they felt themselves to be well-prepared to manage overweight and obese patients compared to those who were working in a chain pharmacy. In addition, they provided significantly more frequent height measurement, BMI calculation, and patient information materials than their counterparts working in a chain pharmacy. Compared with independent pharmacies, the structure of chain operations tends to be more complicated, and CPs working in chain pharmacies may have less flexibility and decision-making capacity and a less personal relationship with their customers. Furthermore, they may need to devote a significant amount of time on non-clinical services, and thus may not spend as much time in patient-centered services such as WMS. The facilities available in the pharmacy may also limit the ability of CPs working in a chain pharmacy to deliver the WMS as desired. For instance, CPs who were working in a chain pharmacy had a stronger perception on the barrier related to a lack of private consultation room than their counterparts working in an independent pharmacy.

In terms of age, CPs who were aged less then 30 years expressed significantly stronger agreement that medication adherence is beneficial for weight loss compared to those CPs between 41–50 years. Although the years of experience in community pharmacy practice among the respondents in our study was unknown, age could be an indicator of years of experience. CPs aged between 41 and 50 years, who have presumably more years of experience in pharmacy practice, may be more confident in achieving positive weight outcomes with lifestyle modification counseling without the need for weight loss medication, compared to those aged less than 30 years. Moreover, CPs who were more senior may have more experience in performing sophisticated body fat percentage measurement.

It is interesting to note that CPs who were holding a bachelor’s degree of pharmacy performed significantly more frequent weight measurement and BMI calculation than CPs who were holding a master’s degree. A survey among Scottish CPs found that a lower proportion of them received weight management training during their postgraduate degree compared to undergraduate pharmacy degree (Newlands et al., 2011). In addition, it may be possible that CPs who were holding a master’s degree in our study undertook a postgraduate program not related to pharmacy or public health, which places little emphasis on weight management.

### Strengths and Limitations of the Study

To the best of the authors’ knowledge, this was the first study that looked at CPs’ involvement in WMS in Malaysia, and to evaluate the Malaysian CPs’ perception on WMS in detail. The findings provide an indication of CPs’ attitudes on weight management in the South-East Asia region and provide scope for further research in this area.

However, several limitations of the study should be noted. Since the study utilized a self-administered questionnaire, there could be a possibility that the respondents intentionally provide a positive response. The study was also limited by cross-sectional nature of data as changes in respondents’ perceptions over time could not be detected. In addition, the cross-sectional design of the study limited our ability to draw valid conclusions on causal relationships between sociodemographic characteristics and attitudes, practices and perceived barriers of CPs on WMS. Another limitation was that some responses given by participants were dependent on their ability to recall experiences with their patients. The finding of this study might also not be generalizable to all Malaysian CPs as only six states in Malaysia were included.

### Recommendations

This study could be taken as a baseline study on Malaysian CPs’ perceptions on WMS. Although our findings revealed that Malaysian CPs generally had a positive perception toward WMS, there is still room for improvement, especially with regards to performance of anthropometric and physiological measurements. Ideally, every CP should perform such measurements to obese and overweight patients who seek for pharmacist’s advice. Professional associations such as MPS can provide training to their members and equip them with up-to-date weight management knowledge to better serve the patients. To ensure standard practices across all community pharmacies that provide WMS, an accreditation process should be implemented specifically for WMS. The obesity epidemic in the country has indirectly proven the fact that the current weight management measures are far from satisfactory which gives CP the opportunity to be at the forefront of addressing this public health concern. While the authorities do have their roles to play in the transformation of health care system which entrusts the CPs to participate in patient-centered public health activities, CPs themselves should shift their professional responsibilities accordingly, from merely dispensing to providing extended pharmacy services such as weight management. Instead of indulging in price war within the competitive pharmaceutical market which would reduce the profit margins, participation in extended pharmacy service of weight management may be an innovative means to generate extra revenue for the community pharmacy. Future research could focus on the perception of Malaysian general public toward the delivery of WMS in community pharmacy to identify opportunity for improvement of such service.

## Conclusion

The study showed that Malaysian CPs generally had a positive perception toward WMS. CPs generally perceived themselves to be well-prepared for the delivery of WMS. The most common barriers noted were a lack of staff to provide WMS and ethical and legal issues related to products/drugs used in obesity management. A few practice and sociodemographic characteristics such as age group, type of pharmacy, highest education qualification, and employment status of CPs were found to influence the attitudes, practices and barriers associated with WMS.

## Availability of Data and Materials

The datasets used and/or analyzed during the current study are available from the corresponding author on request.

## Author Contributions

RV is a Ph.D. candidate and designed the methodology, conducted the study, and wrote the initial version of the manuscript and performed subsequent revisions. WC, NT, and TP are the Ph.D. supervisors and co-supervisors, respectively, and helped in finalizing the methodology, literature review, and in conducting the study, and commented on the initial version of the manuscripts. TP is a member of Pharmacy Board of Malaysia (MPB), Ministry of Health Malaysia. He has conceptualized the research study and recognizes that CPs-led weight management services could be a good platform to serve overweight and obese patients in Malaysia. All authors have reviewed and approved the manuscript.

## Conflict of Interest Statement

The authors declare that the research was conducted in the absence of any commercial or financial relationships that could be construed as a potential conflict of interest.

## Abbreviations

AHLP: A Healthier Life Program
BMI: body mass index
CPs: community pharmacists
IQR: interquartile range
MPS: Malaysian Pharmaceutical Society
SPSS: Statistical Package for the Social Sciences
WHO: World Health Organization
WMS: weight managements services
